# Connectivity in the Dorsal Visual Stream Is Enhanced in Action Video Game Players

**DOI:** 10.3390/brainsci14121206

**Published:** 2024-11-28

**Authors:** Kyle Cahill, Timothy Jordan, Mukesh Dhamala

**Affiliations:** 1 Department of Physics and Astronomy, Georgia State University, Atlanta, GA 30303, USA; kcahill.neurophys@gmail.com; 2 Tri-Institutional Center for Translational Research in Neuroimaging and Data Science (TReNDS), Georgia State University, Georgia Institute of Technology, and Emory University, Atlanta, GA 30303, USA; 3 Department of Psychiatry and Biobehavioral Sciences, University of California Los Angeles, Los Angeles, CA 90095, USA; timjordan.neurophys@gmail.com; 4 Neuroscience Institute, Georgia State University, Atlanta, GA 30303, USA; 5 Center for Behavioral Neuroscience, Georgia State University, Atlanta, GA 30303, USA; 6 Center for Diagnostics and Therapeutics, Georgia State University, Atlanta, GA 30303, USA

**Keywords:** visual information processing, dorsal visual, ventral visual, brain connectivity, video game playing, sensorimotor decision-making

## Abstract

Action video games foster competitive environments that demand rapid spatial navigation and decision-making. Action video gamers often exhibit faster response times and slightly improved accuracy in vision-based sensorimotor tasks. **Background/Objectives:** However, the underlying functional and structural changes in the two visual streams of the brain that may be contributing to these cognitive improvements have been unclear. **Methods:** Using functional and diffusion MRI data, this study investigated the differences in connectivity between gamers who play action video games and nongamers in the dorsal and ventral visual streams. **Results:** We found that action video gamers have enhanced functional and structural connectivity, especially in the dorsal visual stream. Specifically, there is heightened functional connectivity—both undirected and directed—between the left superior occipital gyrus and the left superior parietal lobule during a moving-dot discrimination decision-making task. This increased connectivity correlates with response time in gamers. The structural connectivity in the dorsal stream, as quantified by diffusion fractional anisotropy and quantitative anisotropy measures of the axonal fiber pathways, was also enhanced for gamers compared to nongamers. **Conclusions:** These findings provide valuable insights into how action video gaming can induce targeted improvements in structural and functional connectivity between specific brain regions in the visual processing pathways. These connectivity changes in the dorsal visual stream underpin the superior performance of action video gamers compared to nongamers in tasks requiring rapid and accurate vision-based decision-making.

## 1. Introduction

Action video games immerse the gamer in a fast-paced, challenging environment that heavily taxes vision-based decision-making. Gamers must rapidly process visual information and make swift yet accurate decisions, often refining their skills through many hours of dedicated play. While the psychology and neuroscience literature have reported negative effects of video game playing such as the potential for video game addiction [1,2,3] and increased aggression in those who play violent video games [4,5], there are also cognitive benefits of video game playing that are well documented. Prolonged exposure to action video games is associated with improved cognitive performance, ranging from improvements in low-level perceptual decision-making to high-level cognitive flexibility [6,7,8,9,10,11,12,13,14]. Action video gamers demonstrate better filtering of distracting information [15], enhanced spatial attention [16], improved accuracy in tracking multiple objects [17], enhanced visual search [18,19], reduced sensitivity to backward masking [20], improved contrast sensitivity [21], and mental rotation skills [22]. In addition to these cognitive benefits, neuroimaging studies have demonstrated various structural and functional alterations in certain brain areas and associated brain networks among action video gamers [23,24,25,26,27,28,29,30,31,32,33]. However, only a few video game studies have conducted analysis on both structural and functional MRI data and even fewer have done so in healthy, non-addicted participants [25,33]. Additionally, many previous studies, including those involving video game interventions, have not demonstrated how changes in neuroimaging metrics are reflected in cognitive changes due to a lack of cognitive tests in these studies to ascertain the relationship between cognitive function and neuroplastic changes [33]. This leaves results open-ended, as it is inconclusive whether the neuroimaging changes found in one study can predict behavioral changes found in other studies and whether functional or structural measures are more effective as potential biomarkers.

The human brain is widely recognized as an intricate, complex biological system. A complete understanding of how it operates and adapts requires functional and structural analysis of its constituent subsystems [34,35]. Neuroplasticity, the brain’s ability to reorganize its structure and function in response to stimuli such as action video game play, is thought to underlie these cognitive and behavioral improvements, with alterations in both structural and functional connectivity serving as measurable markers of this adaptive process. Establishing clear brain–behavior relationships, such as long-term action videogame playing and the neuroplastic changes it induces, addresses a significant knowledge gap between behaviors and induced brain network-level changes. These neuroplastic changes may contribute to improvements in behavioral metrics [36] associated with cognitive processes, such as response times during vision-based sensorimotor decision-making. This highlights two important brain–behavior relationships: the connection between behaviors inducing neuroplastic changes, and the neuroplastic changes driving improvements in behavioral observables associated with specific cognitive processes due to engaging in those behaviors.

To help address this gap in the literature, this study investigates how long-term action video game play impacts both functional and structural connectivity in the two visual streams—the dorsal “how” stream and the ventral “what” stream—which are the networks most closely involved in visual processing as outlined by the well-established “two-streams” hypothesis [37,38,39,40,41,42,43,44,45]. In a previous study using a low-level moving-dot task designed to probe participants’ vision-based sensorimotor decision-making accuracy and response time, we found that action video gamers have faster response times (~190 ms) without compromising accuracy [13,46]. By examining the two visual streams, we aim to expand the current understanding of the impact that long-term action video game playing has on the neural mechanisms driving this improvement in vision-based sensorimotor decision response times by illuminating clear brain–behavior relationships between parts of the streams that show significant differences in connectivity metrics between gamer and nongamer cohorts. These brain–behavior relationships would potentially provide an effective biomarker that predicts a person’s vision-based sensorimotor decision-making response time.

In this study, we utilized both functional connectivity and structural connectivity measures. We used undirected functional connectivity (FC), also called a Pearson correlation, and a directed functional connectivity measure, time domain Granger causality (TGC). As opposed to undirected FC analysis, TGC allowed for the determination of the directional influence of one region onto another. This method elucidates how visual information is processed through both visual streams and how this flow of information is altered for long-term game-playing. From both measures, we were able to establish not only brain–behavior relationships with these functional measures but also determine if the directionality of information flows plays an important role in the observed behavioral benefits.

Our structural connectivity analysis used fractional anisotropy (FA), a standard diffusion tensor imaging measure based on diffusivity [44] that has known associations with “action per minute” in certain brain areas of participants who underwent training through playing the real-time strategy game “Starcraft II”, including the left inferior longitudinal fasciculus [29]. Additionally, we considered the quantitative anisotropy (QA) measure because QA-based tractography is known to outperform FA-based tractography and is derived from the Fourier transform relation between the MR signal and diffusion displacement [47]. Utilization of both of these measures allowed for a clear and intuitive characterization of the white matter organization and microstructures by leveraging diffusivity and diffusion displacement.

Given that action video games involve extensive spatial exploration, navigation, and rapid time coordination, we hypothesize that both functional and structural connectivity within the visual streams may undergo neuroplastic enhancements due to prolonged action video game playing. Furthermore, we posit that elevated brain connectivity metrics are likely related to the improved response times observed in gamers. This study addresses changes in neuroimaging metrics due to action video game playing and conclusively determines the effectiveness of each metric in predicting response times during vision-based sensorimotor decision-making. By employing these neuroimaging methods, we aim to enhance the understanding of how long-term action video game playing affects connectivity in the visual streams and its relationship with response time, while also providing a framework for subsequent research in this field.

## 2. Materials and Methods

### 2.1. Subject Data

A total of 47 right-handed participants (28 gamers (4 female) and 19 nongamers (12 female)) were recruited to participate. Groups were age-matched (gamers = 20.6 ± 2.4 years; nongamers = 19.9 ± 2.6 years). Participants who indicated playing 5 h per week or more in one of four types of video game genres for the last two years were considered video game players, i.e., gamers. The four types of action video game genres we recruited, based on industry demographics, were First-Person Shooter (FPS), Real-Time Strategy (RTS), Multiplayer Online Battle Arena (MOBA), and Battle Royale (BR) players. Participants who were nongamers in this study averaged less than 30 min per week in any video game over the last two years. A modified left–right moving-dot (MD) motion categorization task was used to probe for differences in reaction speed and accuracy between the cohorts [13,27]. We excluded one gamer from the structural connectivity analysis due to incomplete tractography data, and one nongamer from the functional connectivity analysis due to incomplete fMRI data. Two additional subjects’ data (one gamer and one nongamer) were excluded from the brain–behavior regressions due to incomplete response time data. The effective number of participants for the functional connectivity analysis was 46 total participants (28 gamers and 18 nongamers) with 44 total participants (27 gamers and 17 nongamers) for brain–behavior regression between functional connectivity measures with response time. The effective number of participants for the structural connectivity analysis was 46 total participants (27 gamers and 19 nongamers) with 44 total participants (26 gamers and 18 nongamers) for brain–behavior regression between structural connectivity measures with response time.

### 2.2. MRI Data

Whole-brain structural and functional MR imaging was conducted on a 3 T Siemens Magnetom Prisma MRI scanner (Siemens, Atlanta, GA, USA) at the joint Georgia State University and Georgia Institute of Technology Center for Advanced Brain Imaging, Atlanta, GA, USA. First, high-resolution anatomical images were acquired for voxel-based morphometry and anatomical reference using a T1-MEMPRAGE scan sequence (TR = 2530 ms; TE1-4: 1.69–7.27 ms; TI = 1260 ms; flip angle = 7 deg; voxel size 1 mm × 1 mm × 1 mm). Following, four functional runs used a T2*-weighted gradient echo-planar imaging sequence (TR = 535 ms; TE = 30 ms; flip angle = 46°; field of view = 240 mm; voxel Size = 3.8 mm × 3.8 mm × 4 mm; number of slices = 32, collected in an interleaved order; slice thickness = 4 mm) and acquired 3440 brain images while participants performed the behavioral tasks [13,27].

### 2.3. Regions of Interest

Our study focuses on the dual streams of the primary visual cortex because these regions are directly involved in processing visual information and guiding visuomotor behavior, which is central to our task involving moving visual stimuli. The dorsal stream and the ventral stream are both highly relevant for understanding how the brain integrates visual and motor information. Thus, our choice of ROIs reflects the specific neural mechanisms underpinning the task we investigated, rather than those associated with higher-order, reward-based decision-making.

**Figure 1:**
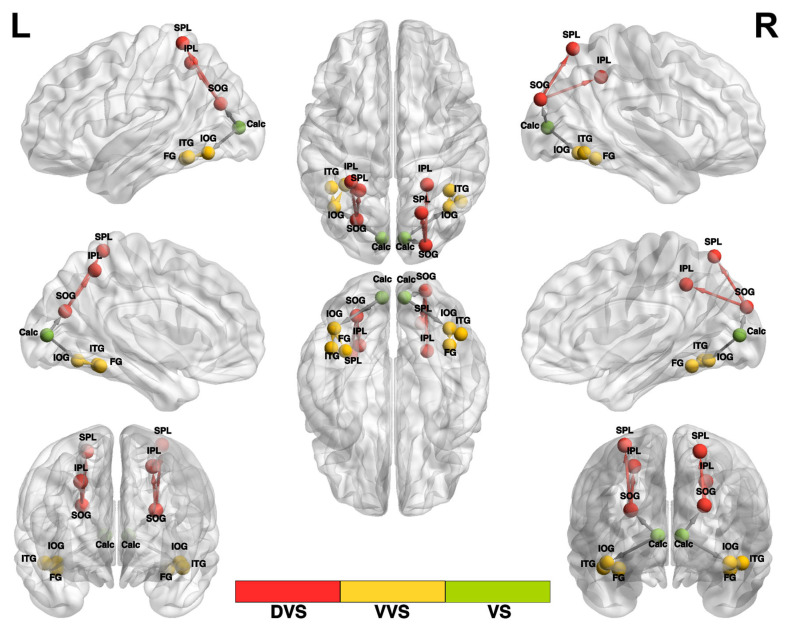
Meta-analysis-derived ROIs for the dorsal and ventral visual streams. BrainNet Viewer representation of the locations of the 14 spherical ROIs and 12 connections considered that constitute the subsystems of the two visual streams. (**Left** to **Right**): The dorsal visual stream (**DVS**) extends from SOG to IPL and from SOG to SPL, shown in red; the ventral visual stream (**VVS**) extends from IOG to FG and from IOG to ITG, shown in yellow; and the visual stream (**VS**) denotes the primary connections from the calcarine region, namely, calcarine to IOG and calcarine to SOG.

In our investigation, we examined the structural and functional organization of the dorsal and ventral visual streams using fourteen regions of interest (ROIs) defined in a prior study from the Neurosynth functional meta-analysis platform (https://neurosynth.org/) with their MNI coordinates denoted in Table 1. The ROIs were derived based on relevant search terms, including “primary visual,” “ventral visual,” “visual stream,” and “dorsal visual” [47]. The identified ROIs encompassed two regions situated within the primary visual cortex (V1), specifically the bilateral calcarine (Calc) areas. VS (visual stream) denotes the primary connections from the calcarine region to the bilateral superior and inferior occipital gyri. Additionally, we identified four ROIs within the ventral visual stream (VVS), which included the bilateral fusiform gyrus (FG) and inferior temporal gyrus (ITG). Moreover, four ROIs were identified in the dorsal visual stream (DVS), encompassing the bilateral inferior parietal lobule (IPL) and superior parietal lobule (SPL). The nomenclature used in this classification was based on the Eickhoff–Zilles macro-labels from N27 and was implemented in AFNI [47]. We constructed a BrainNet Viewer [48] representation of the general organization, subsystems, and the 14 ROIs along with the 12 connections composing the visual streams (Figure 1). 

The construction of the ROIs for the structural tractography was carried out with a 12 mm radius to ensure the analysis accounted for anatomical variability and adequately encompassed the white matter tracts connecting the visual streams, thus mitigating the risk of missing important connections. We employed the MNI coordinate system and constructed the ROIs using the FSLeyes visualization tool within the FSL version 6.0.6 (FMRIB Software Library) environment. The functional connectivity analysis utilized 6 mm radius ROIs using the same MNI coordinates.

Statistical comparison of connectivity metrics between ROIs across gamers and nongamers was carried out using the Wilcoxon rank-sum test (also known as the Mann–Whitney U test). This nonparametric test was chosen because it does not assume normality of the connectivity metrics’ distribution, making it suitable for comparing the two groups without requiring any assumptions about the underlying distribution of the data. For multiple comparison corrections in this analysis, we employed the Holm–Bonferroni method. This method was selected for its ability to enhance statistical power and sensitivity to individual significant comparisons while effectively controlling for Type I errors [49,50]. We applied a significance threshold of *p* < 0.05, with the Holm–Bonferroni correction indicated by p*, within each main section (DVS, VVS, and VS) to ensure statistical significance while simultaneously controlling for the family-wise error rate in the dorsal and ventral streams, providing a robust framework for identifying meaningful differences in connectivity metrics.

### 2.4. Functional Connectivity Protocols and Analysis

Functional connectivity in neuroimaging measures temporal correlations of the BOLD signal time series in spatially distant brain regions [51,52,53]. Thus, two regions are considered functionally connected if there is a statistical relationship between the measures of recorded BOLD activity [54]. Region- and task condition-specific fMRI time series segments were normalized, voxel-averaged, and corrected for linear trends. To assess each participant’s region-to-region undirected functional connectivity (FC), pairwise Pearson correlation coefficients were computed using 6 mm spherical ROIs. The appropriate model order for the Granger causality (GC) analysis was determined by minimizing the spectral difference between the Granger time series and the original time series. After evaluating model orders ranging from two to twenty, a model order of six was selected, as it best minimized the spectral difference in our dataset. GC matrices were then computed using this optimal model order.

The GC from region 2 to region 1 in the frequency domain is defined as follows:

$$I_{2\to 1}\left(f\right)=\ln\left(\frac{S_{11}\left(f\right)}{S_{11}\left(f\right)-\left(\sum_{22}-\frac{{\sum_{12}}^{2}}{\sum_{11}}\right){\left|H_{12}(f)\right|}^{2}}\right)$$

The frequency band-specific or time domain-equivalent GC is as follows:

$$F_{2\to 1}\left(f\right)=\frac{1}{f_{2}-f_{1}}\int_{f_{1}}^{f_{2}}I_{2\to 1}\left(f\right)df$$

In the bivariate autoregressive model [55,56,57], the noise covariance and transfer function matrices are denoted by “∑” and “*H*”, respectively. The evaluation of the frequency band-specific or time domain-equivalent Granger causality (GC) was accessed in the range between $f_{1}$ = 0.05 Hz and $f_{2}$ = 0.9 Hz, with a sampling rate of 1.87 Hz ($TR^{-1}$).

To investigate the relationship between brain connectivity and behavior, undirected FC values and directed time domain Granger causality (TGC) values were obtained for each participant per connection. Spearman’s rank correlation coefficient was used to assess the correlation between FC and reaction time (RT) as well as between TGC and RT.

### 2.5. Tractography Protocols and Structural Connectivity Analysis

DSI Studio version 2022.08.0 is a non-commercial software program that was utilized in this study for diffusion MR image analysis and provided functions including deterministic fiber tracking and 3D visualization [58]. In this study, we used a multi-shell diffusion scheme with b-values of 300, 650, 1000, and 2000 s/mm². The acquisition parameters consisted of an in-plane resolution of 2 mm and a slice thickness of 2 mm. The accuracy of b-table orientation was examined by comparing fiber orientations with those of a population-averaged template [59]. The diffusion data were reconstructed in the MNI space using q-space diffeomorphic reconstruction [60] to obtain the spin distribution function [58,61]. A diffusion sampling length ratio of 1.25 was used. The resulting diffeomorphic reconstruction output had an isotopic resolution of 2 mm. Tensor metrics were calculated and a deterministic fiber tracking algorithm was used to track the fiber pathways.

Seeds were randomly placed throughout the ROIs until reaching a cutoff at 50,000,000 seeds. Additionally, two pairwise spherical ROIs were also defined as ending regions. In the case between the left superior occipital gyrus (L SOG) and the left inferior parietal lobule (L IPL), for example, the ending regions were placed at (−26,−73,23) and (−24,−52,52). An angular threshold of 60 degrees was set as the maximum allowed angular deviation between steps. The step size was randomly selected from 0.5 voxels to 1.5 voxels. Tracks with lengths shorter than 10 mm or longer than 100 mm were excluded from further analysis.

**Figure 2:**
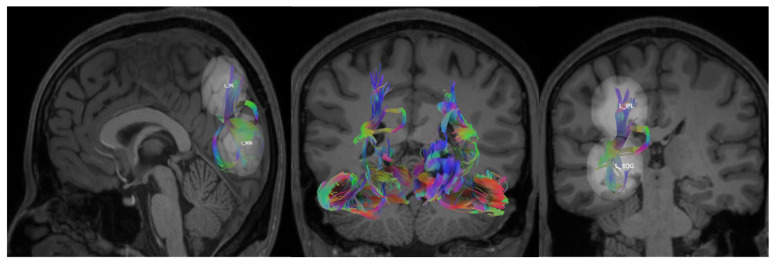
L SOG—L IPL fiber tracts overlayed on a T1 image in a representative participant. The fibers are colored-coded using the RGB model to represent their orientation, where “**red**” indicates fibers along the X-axis (i.e., left–right), “**green**” indicates fibers along the Y-axis (i.e., anterior–posterior), and “**blue**” indicates fibers along the Z-axis (i.e., inferior–superior). Mixed colors, such as “**yellow**”, represent fibers with combined orientations (e.g., red and green). (**Left to Right**): **sagittal**: reconstruction of white matter fiber tracts modeling pathways between L SOG and L IPL; **coronal**: reconstruction of white matter tracts modeling the pathways that constitute the entire dorsal and ventral streams; **coronal**: reconstruction of white matter tracts modeling the pathways between the L SOG and L IPL.

The process continued until mapping each subsystem of the dorsal and ventral visual streams (DVS, VVS, and VS), with an exhaustive exploration of all pairwise links in each section. Adjustments to the parameters for maximum length and angular threshold were made based on the connection being mapped, as detailed in Table 2. For tracking eligibility, a quantitative anisotropy threshold of 0.01 was universally applied in all connections, except for the connection between R SOG and R IPL. In this case, a lower quantitative anisotropy threshold of 0.005 was necessary to prevent the inadvertent exclusion of subjects with valid fibers from the analysis. Consequently, voxels with a qualitative anisotropy value exceeding the specified threshold were deemed anisotropic and deemed suitable for inclusion in the fiber tracking process. The fiber pathways between the L SOG and L IPL are shown in a representative subject in Figure 2 where the axis is color-coded to distinguish the orientation of the fibers. The X-axis is coded for red from right to left, the Y-axis is coded for green from anterior to posterior, and the Z-axis is coded for blue from superior to inferior.

Structural connectivity measures the anatomical organization of the brain using white matter fiber tracts [62]. Although relatively stable on shorter time scales (seconds to minutes), it can exhibit plastic experience-dependent changes at longer periods (hours to days) [63]. Fractional anisotropy (FA), based on diffusivity, is calculated as the normalized fraction of the diffusion tensor’s magnitude [64]. Fractional anisotropy (FA) is a useful measure and is often a standard in structural connectivity analysis [64,65]. 

Quantitative anisotropy (QA) is a model-free measure derived from the Fourier transform relation between MR signals and diffusion displacement and is nonparametrically calculated from peak orientations on a spin distribution function [58]. QA is known to be less susceptible to partial volume effects due to crossing fibers and free water in the brain than FA, resulting in a better resolution and improved tractography [60,66]. Both fractional anisotropy (FA) and quantitative anisotropy (QA) were utilized as primary measures of enhanced structural connectivity and were extracted for each pair of ROIs composing the two visual streams, and we investigated the brain–behavior relation of these two structural connectivity metrics with the participants’ response times.

## 3. Results

### 3.1. Functional Connectivity

#### 3.1.1. Elevated Functional Connectivity in Gamers and Correlation with Response Time

**Figure 3:**
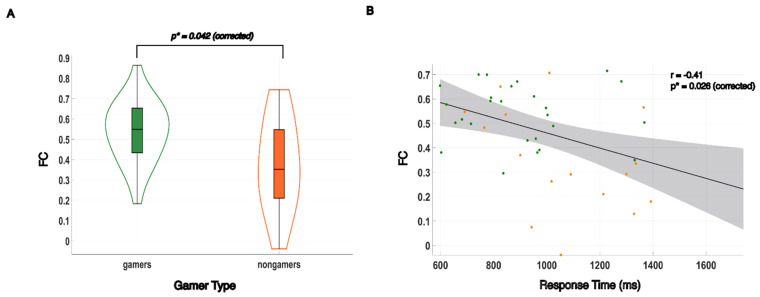
Undirected functional connectivity between the left superior occipital gyrus and left superior parietal lobule and brain–behavior correlation with response times. (**A**) Gamers showed significantly higher FC values between the L SOG and the L SPL, p* = 0.042. (**B**) The undirected functional connectivity measure was significantly correlated with response time, (r = −0.41, p* = 0.026). Gamers are indicated by green dots while nongamers are indicated by orange dots.

Pairwise Pearson correlation was utilized for undirected functional connectivity analysis. Statistical comparison between video gamers and nongamers was conducted using the Wilcoxon rank-sum test. In comparison, gamers exhibited higher FC values between the L SOG and the L SPL. This result survived Holm–Bonferroni multiple comparison correction after accounting for four connections among the ROIs composing the dorsal stream and remained statistically significant at the 0.05 level, p* = 0.042. A violin plot was constructed to graphically illustrate the significant difference found in FC values between gamers and nongamers in Figure 3A. FC values were plotted against RT, and a linear regression analysis, depicted in Figure 3B, was employed to explore the existence of a brain–behavior link; FC was found to have a significant moderate level correlation with subjects’ response time (r = −0.41, p* = 0.026).

#### 3.1.2. Elevated Granger Causality in Gamers and Correlation with Response Time

**Figure 4:**
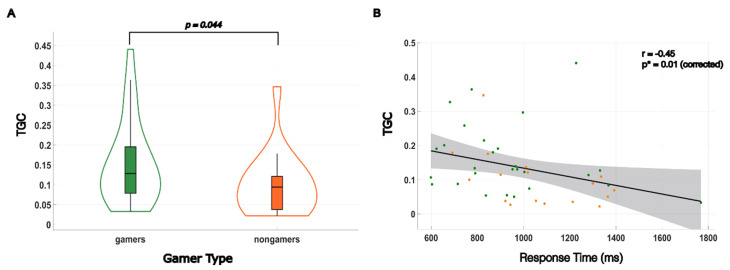
Directed functional connectivity (TGC) between the left superior occipital gyrus (L SOG) and left superior parietal lobule (L SPL) and brain–behavior correlation with response times. (**A**) Gamers showed significantly higher TGC values between the R SOG and the R SPL, uncorrected *p* = 0.044. (**B**) The directed functional connectivity measure TGC was significantly correlated with response time (r = −0.45, p* = 0.01). Gamers are indicated by green dots while nongamers are indicated by orange dots.

The directed connectivity analysis utilized pairwise Granger causality to determine differences in directional influence along the visual streams. After testing a range of possible model orders through computation aimed at minimizing the total spectral difference between the Granger time series and the original, we found that the optimal model order for our dataset was a model order of six. Time domain Granger causality (TGC) was computed for all pairwise links between subjects. Comparing gamers to nongamers, elevated TGC values were also observed between the L SOG and the L SPL with an uncorrected *p*-value, *p* = 0.044. This significant difference in TGC values is visually represented in Figure 4A, through a violin plot. We further investigated the relationship between time domain Granger causality values and observed response times. TGC values were plotted against RT, and a linear regression analysis, depicted in Figure 4B, was employed to explore the existence of a brain–behavior link; TGC was found to have a significant moderate level correlation with subjects’ response time (r = −0.45, p* = 0.01 (corrected)).

### 3.2. Structural Connectivity

#### Elevated Fractional Anisotropy and Quantitative Anisotropy in Gamers

**Figure 5:**
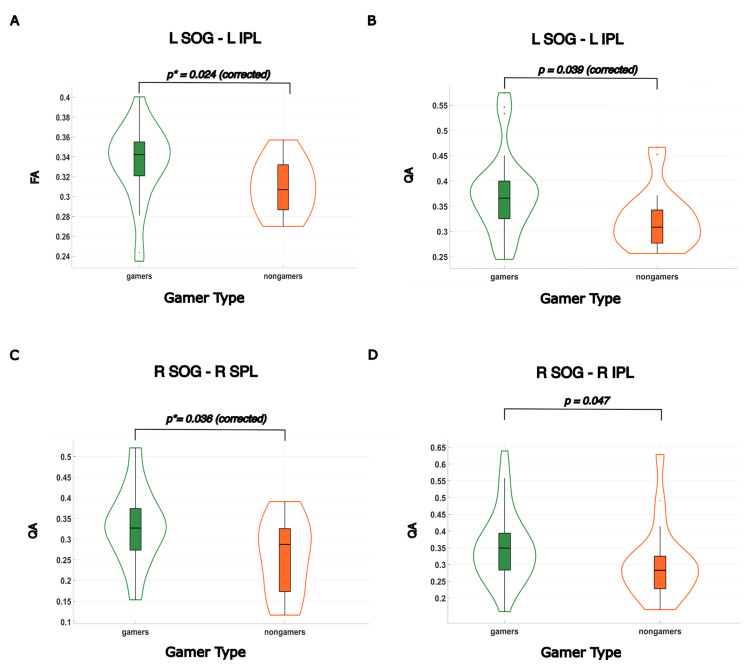
Structural connectivity in the dorsal stream is elevated in gamers. (**A**) Gamers showed significantly higher FA values between the left superior occipital gyrus (L SOG) and the left inferior parietal lobule (L IPL), p* = 0.024. (**B**) Video gamers showed significantly higher QA values between the L SOG and the L IPL, *p* = 0.039. (**C**) Video gamers showed significantly higher QA values between the right superior occipital gyrus (R SOG) and the right superior parietal lobule (R SPL), p* = 0.036. (**D**) Video gamers showed significantly higher QA values between the R SOG and the right superior parietal lobule (R IPL), uncorrected *p* = 0.047.

The statistical comparison of structural connectivity measures, specifically, fractional anisotropy and quantitative anisotropy between gamers and nongamers, utilized the Wilcoxon test. In this analysis, gamers exhibited elevated FA values between the L SOG and the L IPL (p* = 0.024). Gamers also demonstrated elevated QA values between the same regions (L SOG and L IPL) with statistical significance (p* = 0.039). In addition to the observed elevation in FA and QA values within the left dorsal stream, notably between L SOG and L IPL, our investigation revealed heightened QA values in the right dorsal stream as well. Specifically, gamers exhibited increased QA R SOG and the R SPL (p* = 0.036), as well as between R SOG and the R IPL (*p* = 0.047). The increased QA between R SOG and R IPL did not survive multiple comparison corrections but remained significant at the individual level, warranting consideration for further study. To visually illustrate the significant differences in values between gamers and nongamers, violin plots were constructed (Figure 5). Structural connectivity measures FA and QA did not show a significant correlation with subjects’ response time.

## 4. Discussion

This study investigated functional and structural connectivity differences between gamers and nongamers in the dorsal and ventral visual streams. From our structural results, we observed that gamers exhibited increased white matter structural connectivity within the dorsal visual stream. Specifically, the connection in the left DVS between the left superior occipital gyrus and the left inferior parietal lobule showed elevated fractional anisotropy (FA) and quantitative anisotropy (QA) values, as well as elevated QA values in the right DVS. FA has known associations with axonal integrity and myelination, while QA is linked to axonal density [67,68,69]. These properties could indicate greater directional coherence and suggest better white matter fiber tract organization between brain areas. Although structural differences were observed, they did not correlate with response time.

We also observed higher functional connectivity values in the left DVS for gamers compared to nongamers. We found that undirected functional connectivity values and time domain Granger causality values were higher between the left superior occipital gyrus and the left superior parietal lobule. Both measures of connectivity showed moderate correlations with response times, reflecting that they may play a strong role in influencing gamer performance, specifically in response times. Taken together, these results show that extensive video game playing does alter both structural and functional connectivity in gamers, which supports previous findings. However, our study shows that the functional connectivity measures FC and TGC serve as better gauges of participants’ response times compared to the structural connectivity measures FA and QA during vision-based sensorimotor decision-making. While the elevated TGC values did not survive corrections for multiple comparisons, this should be further explored in future studies due to its correlation with performance. And because the TGC measure allowed for us to determine the directional influence of one region on another, we were able to assess how the directional flow of visuospatial information is altered by long-term game playing. Our findings suggest that the efficiency of information processing within the dorsal visual stream may contribute to the faster decision-making and response times observed in gamers, without compromising accuracy.

This study has some limitations, including an imbalance in the sex distribution between cohorts [70] and the focus on healthy, age-matched young adults [71,72,73,74], which could potentially influence the generalizability of our findings. Future research should address these limitations with a more diverse participant group. While our study’s sample size was sufficient for statistical comparison, larger participant pools in future studies would increase overall statistical power to further investigate subgenre-specific effects, account for intrasubject variability more robustly, and ultimately improve generalizability by employing techniques such as logistic regression and mixed effects models. Incorporating perfusion MRI in future studies could provide additional validation of our findings by linking observed connectivity changes to changes in cerebral blood flow (CBF), offering a complementary physiological perspective. There are also limitations of anisotropy analysis in assessing structural connectivity due to limitations in spatial resolution and the indirect nature of anisotropy measures. Additionally, due to voxel-level averaging, diffusion measures may be affected by partial volume effects, particularly in areas with crossing fibers, complex fiber orientations, or the presence of free water in the brain. FA specifically has limitations in that this measure is known to suffer from these potential confounds [58]. Consideration of these known weaknesses in FA-based tractography informed our decision to implement QA-based tractography to conduct the structural connectivity analysis since QA-based tractography is less susceptible to these confounding effects while still recording DSI Studio’s estimation of FA between ROIs [58]. Lastly, this study combined all gamers into one group instead of separating them into their respective genres. Future research should continue to explore the impact of specific games, game genres, and subgenres on specific cognitive processes and their effects on brain connectivity to better understand the mechanisms driving cognitive improvements similar to some previous studies [75,76].

A crucial aspect of advancing our understanding of neuroplasticity due to video game playing involves continued research into both the rate at which structural and functional connectivity changes occur among gamers and the identification of key factors that may influence this rate. Longitudinal studies, tracking connectivity changes over time, could provide valuable insights into the timeline of these changes in response to gaming experiences, and the relevant factors influencing this timeline could reveal critical thresholds and strategies for optimizing skill acquisition and windows for skill transfer [77,78]. Furthermore, future work should consider alterations in the network topology of brain structural networks in gamers versus nongamers. Changes in the connection structure—such as a shift from random to more efficient small-world or scale-free network configurations—could provide deeper insights into brain network plasticity [79]. This approach could complement the structural and functional connectivity findings from our study by offering a broader perspective on how long-term gaming in various game genres may reorganize brain networks [79]. The analysis presented in this study can be extended to investigate possible connectivity differences in interstream interactions between gamers and nongamers. Previous work suggests that interactions between the dorsal and ventral streams are necessary for skilled grasp [80] and complex object manipulation [81], which gamers engage in frequently while playing an action video game. The analysis presented in this study can be extended to investigate possible connectivity differences in interstream interactions between gamers and nongamers. Finally, we plan to expand the functional and structural connectivity methods implemented in this study to the whole brain and expand upon our understanding of brain-wide neuroplastic changes due to long-term exposure to action video game playing.

Our findings offer robust evidence of enhanced structural and functional connectivity within the dorsal visual stream, highlighting the influence of video game experience on neuroplasticity. The dorsal stream is crucial for visuomotor integration, and since we screened for right-handed participants in this study, this could explain why functional connectivity in this part of the left DVS is elevated in gamers given the standard lateralization of the brain, and why functional connectivity measures in this region are significantly linked with response times. Prior research has established the dorsal stream’s involvement in tracking object location and trajectories in space [38], which is vital information in action video games. The increased functional connectivity measures FC and TGC between the left superior occipital gyrus (L SOG) and the left superior parietal lobule (L SPL) correlate with improved sensorimotor decision-making response times, indicating that this connection within the “how” pathway is linked to better functional visual information processing, thereby likely driving improvements in response times during vision-based sensorimotor decision-making.

These action video game-driven neuroplastic changes likely extend beyond brain networks to the sensorimotor systems responsible for rapid decision-making and coordination. The concept of embodied cognition, which emphasizes the deep connection between cognitive processes and the body’s interactions with the environment, offers a potentially more comprehensive understanding of these changes [82]. Specifically, the observed enhancements in response times and sensorimotor decision-making in gamers may reflect adaptations in brain connectivity, motor circuits, and sensory feedback mechanisms driven by mechanisms of synaptic plasticity. For instance, Hebbian plasticity, long-term potentiation (LTP), and other forms of synaptic plasticity could facilitate more efficient communication between brain regions and motor circuits, enhancing coordination and decision-making [83]. Additionally, the inhibition or pruning of less efficient pathways may also contribute to the optimization of neural networks, reinforcing more effective neural circuits while reducing the reliance on suboptimal ones. This suggests that video game studies could serve as a valuable platform for testing embodied cognition-inspired hypotheses and the various forms of synaptic plasticity that drive macroscopic changes in connectivity metrics. Such research could broaden our understanding of how interaction with video game environments influences cognition and motor function.

## 5. Conclusions

In summary, this study provides compelling evidence that prolonged action video game exposure enhances both structural and functional connectivity in the dorsal visual stream. Specifically, the observed improvements in functional connectivity between the left superior occipital gyrus and the left superior parietal lobule correlate with faster response times during sensorimotor decision-making tasks. These results underscore the potential of video game experience to drive meaningful neuroplastic changes that enhance cognitive functions such as decision-making and sensorimotor processing. The practical implications of these findings are significant, offering new opportunities for developing targeted cognitive training and educational programs. The demonstrated capacity of video games to promote neuroplasticity suggests their potential utility in designing tailored interventions to strengthen attention, visuomotor coordination, and reaction times in non-clinical populations. This approach could benefit various fields, such as sports, professional training, or education, where rapid decision-making and efficient sensorimotor responses are crucial [84]. Moreover, the dual-metric framework employed in this study, which integrates both structural and functional connectivity measures, provides a comprehensive and intuitive method for understanding how behavioral experiences shape neural networks and influence cognitive performance. The MRI analysis methods used here may be extended to investigate clinical populations, offering a foundation for exploring how targeted interventions, such as video game-based cognitive training, might support rehabilitation efforts in individuals with impaired cognitive or motor functions. This adaptability highlights the broader applicability of our approach and paves the way for future research to test how various video game genres may uniquely influence cognitive processes, expanding the role of video game studies as a valuable tool for investigating and promoting behaviorally induced neuroplasticity [83].

## Figures and Tables

**Table 1:** Regions of interest categorized by subsystem.

Region of Interest	MNI Coordinates x, y, z (mm)	Subsystem
Left superior occipital gyrus (L SOG)	−26, −73, 23	DVS, VS
Left inferior parietal lobule (L IPL)	−24, −52, 52	DVS
Left superior parietal lobule (L SPL)	−30, −46, 66	DVS
Right superior occipital gyrus (R SOG)	23, −91, 26	DVS, VS
Right inferior parietal lobule (R IPL)	24, −48, 42	DVS
Right superior parietal lobule (R SPL)	20, −68, 62	DVS
Left inferior occipital gyrus (L IOG)	−42, −64, −12	VVS, VS
Left inferior temporal gyrus (L ITG)	−44, −50, −15	VVS
Left fusiform gyrus (L FG)	−34, −48, −16	VVS
Right inferior occipital gyrus (R IOG)	40, −64, −12	VVS, VS
Right inferior temporal gyrus (R ITG)	48, −60, −12	VVS
Right fusiform gyrus (R FG)	40, −52, −16	VVS
Left calcarine (L Calc)	−8, −86, 6	VS
Right calcarine (R Calc)	8, −86, 6	VS

**Table 2:** Tracking parameters for connections within each subsystem.

Subsystem	Connection	Min Length (mm)	Max Length (mm)	Angular Threshold (deg)
DVS	L SOG L IPL	10	100	60
	L SOG L SPL	10	300	70
	R SOG R IPL	10	100	75
	R SOG R SPL	10	150	70
VVS	L IOG L ITG	10	20	50
	L IOG L FG	10	35	50
	R IOG R ITG	10	20	50
	R IOG R FG	10	35	50
VS	L Calc L IOG	10	70	65
	L Calc L SOG	5	20	50
	R Calc R IOG	10	80	65
	R Calc R SOG	10	30	50

## Data Availability

All data that support the findings of this study as well as the custom analysis scripts can be found in OSF (https://osf.io/a97gh/).
